# Fructose 1,6-Bisphosphatase 1 Expression Reduces ^18^F-FDG Uptake in Clear Cell Renal Cell Carcinoma

**DOI:** 10.1155/2019/9463926

**Published:** 2019-01-06

**Authors:** Ruohua Chen, Xiang Zhou, Gang Huang, Jianjun Liu

**Affiliations:** ^1^Department of Nuclear Medicine, Ren Ji Hospital, School of Medicine, Shanghai Jiao Tong University, Shanghai, China; ^2^Shanghai University of Medicine and Health Sciences, Shanghai, China

## Abstract

**Purpose:**

To determine the relationship between fructose 1,6-bisphosphatase 1 (FBP1) expression and fluorine 18 (^18^F) fluorodeoxyglucose (FDG) uptake in patients with clear cell renal cell carcinoma (ccRCC), and to investigate how ^18^F-FDG uptake and FBP1 expression are related to tumor metabolism and tumor differentiation grade.

**Materials and Methods:**

A total of 54 patients with ccRCC underwent ^18^F-FDG combined positron emission tomography and computed tomography (PET/CT) before tumor resection. The maximum standardized uptake value (SUVmax) for the primary tumor was calculated from the ^18^F-FDG uptake. The relationship between SUVmax of primary tumor and the expression of FBP1, hexokinase 2 (HK2), and glucose transporter 1 (GLUT1) was analyzed via immunohistochemical analysis.

**Results:**

We identified an inverse relationship between FBP1 expression and SUVmax (*P*=0.031). SUVmax was higher in patients with high-grade ccRCC (mean, 11.6 ± 5.0) than in those with low-grade ccRCC (mean, 3.8 ± 1.6, *P* < 0.001). FBP1 expression was significantly lower in patients with high-grade ccRCC (mean, 0.23 ± 0.1) than in those with low-grade ccRCC (mean, 0.57 ± 0.08; *P*=0.018). FBP1 status could be predicted with an accuracy of 66.7% when a SUVmax cutoff value of 3.55 was used. GLUT1 expression in ccRCC was positively correlated with ^18^F-FDG uptake and FBP1 status, whereas HK2 expression was not.

**Conclusion:**

SUVmax in patients with ccRCC is inversely associated with the expression of FBP1, and FBP1 may inhibit ^18^F-FDG uptake via regulating GLUT1. SUVmax is higher in patients with high-grade ccRCC than in those with low-grade ccRCC, which could be the result of lower FBP1 expression in patients with high-grade ccRCC.

## 1. Introduction

Renal cell carcinoma (RCC) is one of the most prevalent tumors worldwide, with clear cell RCC (ccRCC) as the most common histologic type [1, 2]. Surgical resection remains the most common treatment strategy for RCC [3, 4]; however, approximately 25–30% of RCC patients are diagnosed with metastases, and many cases develop metastases after radical nephrectomy [5–7]. As a result, the overall 5-year survival rate of RCC patients is still poor [4]. Clinicians must make an early diagnosis to improve the survival rate of RCC patients. As a result, the identification of new tumor markers that better reflects the biological characteristics of RCC is of great necessity.

Fluorine 18 (^18^F) fluorodeoxyglucose- (FDG-) combined positron emission tomography (PET) and computed tomography (CT) has been widely used for evaluating tumor activity, which is based on the high rate of glucose metabolism in cancer cells [8–11]. However, previous studies have found that the sensitivity of ^18^F-FDG PET/CT is not very high in the diagnosis of ccRCC because of low ^18^F-FDG uptake in a considerable part of the ccRCC [12–15]. Because ^18^F-FDG PET is increasingly being used as a diagnostic tool in RCC, a further characterization of this phenomenon is essential.

Previous studies have found that the maximum standardized uptake (SUVmax) is higher in high-grade ccRCC than in low-grade ccRCC [15]; however, the molecular mechanisms underlying these findings remain unclear. Glucose homeostasis is reciprocally controlled by anabolic gluconeogenesis and catabolic glycolysis. The kidney is one of the primary sites of anabolic gluconeogenesis, which is next only to the liver. Recently, attention has been mainly focused on the increased glycolysis in tumors, which is called the Warburg effect [16]. However, the possibility that this effect is also being facilitated by altered gluconeogenesis has not been studied.

Fructose 1,6-bisphosphatase 1 (FBP1) is a key enzyme in the gluconeogenesis pathway which catalyzes fructose 1,6-bisphosphate into fructose 6-phosphate [17]. We and others have demonstrated that FBP1 plays an important role in the glucose metabolism of malignant tumors [17–19]. However, the relationship between FBP1 expression and ^18^F-FDG uptake in ccRCC, along with the underlying molecular mechanisms, has not been examined so far.

In the present study, we investigated whether FBP1 expression is associated with ^18^F-FDG uptake in ccRCC and whether ^18^F-FDG uptake can be used to predict FBP1 status. In addition, we examined whether GLUT1 and HK2 expression are associated with ^18^F-FDG uptake in ccRCC by immunohistochemical analysis.

## 2. Materials and Methods

### 2.1. Study Population

This retrospective study included 54 patients with ccRCC (mean age, 58.9 years; age range, 31–82 years); 37 were men (mean age, 57.2 years; age range, 31–77 years) and 17 were women (mean age, 62.6 years; age range, 49–82 years). Patients underwent ^18^F-FDG PET/CT before tumor resection at Ren Ji Hospital between 2009 and 2016. The inclusion criteria were as follows: diagnosis of ccRCC was confirmed by pathologic examination; complete clinical data, including age, sex, tumor size, lymph node metastasis, and tumor grade, were available; tissue specimens for immunohistochemical analysis were available; and follow-up information was available. ccRCCs were classified into 2 categories; tumors containing a nuclear G3 or G4 component and tumors consisting of G1 and G2 components (high- and low-grade ccRCC, respectively). No distant metastasis occurred. Our retrospective study was approved by the Institutional Review Board of Shanghai Jiao Tong University that is affiliated with Ren Ji Hospital, and the requirement to obtain informed consent was waived.

### 2.2. PET/CT Imaging and Analysis

A combined PET/CT device (Biograph mCT; Siemens) was used for all PET/CT scans. PET imaging was carried out with an acquisition time of 3 minutes per bed position after CT scanning. All patients received an intravenous injection of ^18^F-FDG (3.7 MBq/kg) after having fasted for at least 6 h. The mean uptake time was 50 ± 6 min. Blood glucose levels were measured and found to be less than 140 mg/dL at the time the ^18^F-FDG was administered. PET images were iteratively reconstructed, and CT data were used for attenuation correction. For quantitative analysis, two experienced nuclear medicine physicians evaluated ^18^F-FDG uptake on a workstation (Medex) by calculating the SUVmax of the regions of interest. Regions of interest were placed over the suspected lesions that may have exhibited increased ^18^F-FDG uptake. Regions of interest were drawn according to previous contrast-enhanced CT scans in the lesions that exhibited no substantially increased ^18^F-FDG uptake. The SUVmax was calculated as follows: maximum pixel value in the decay-corrected ROI activity (MBq/kg)/(radioactivity of the injected dose (MBq)/body weight (kg)).

### 2.3. Immunohistochemistry

Immunohistochemical staining was performed on paraffin embedded ccRCC tissues. After microtome sectioning (5 *μ*m), the slides were stained with anti-GLUT1 (Proteintech), anti-HK2 (Proteintech), and anti-FBP1 (Sigma) antibodies. Immunohistochemical analyses were conducted by two experienced pathologists. The slides were scored according to staining intensity (0–3). Slides with a score of 2 or 3 were considered high expression, and slices with a score of 0 or 1 were considered low expression.

### 2.4. Statistical Analyses

The data were presented as mean ± SD. The Mann–Whitney *U* test was used to assess the association between SUVmax and FBP1 expression. The relationship between FBP1 expression and clinicopathologic characteristics of RCC patients was assessed by using the Fisher exact or x^2^ test. The Mann–Whitney *U* test was also used to assess the association between the expression of GLUT1 and HK2 and SUVmax. Pearson's rank correlation was applied to determine the association between FBP1 expression and the expression of GLUT1 or HK2. The receiver-operating characteristic curve was used to assess the optimal value of SUVmax for predicting FBP1 expression. *P* < 0.05 was considered significantly different. All statistical analyses were conducted with SPSS software (SPSS, version 13.0).

## 3. Results

### 3.1. Patient Population

Patient characteristics are summarized in Table 1. Among the 54 patients, 37 had low-grade ccRCC and 17 had high-grade ccRCC. Although the majority (82.8%, 39/54) of primary tumors showed positive ^18^F-FDG uptake, 17.8% (15/54) of primary tumors showed negative ^18^F-FDG uptake compared with normal liver tissues. The SUVmax for the primary tumors ranged from 1.7 to 22.1, with an average of 6.21. A total of 46.3% (25/54) of the tumors showed high FBP1 expression, and 53.7% (29/54) of the tumors showed low FBP1 expression.

### 3.2. Correlation between Patient Characteristics, SUVmax, and FBP1 Expression

**Figure 1:**
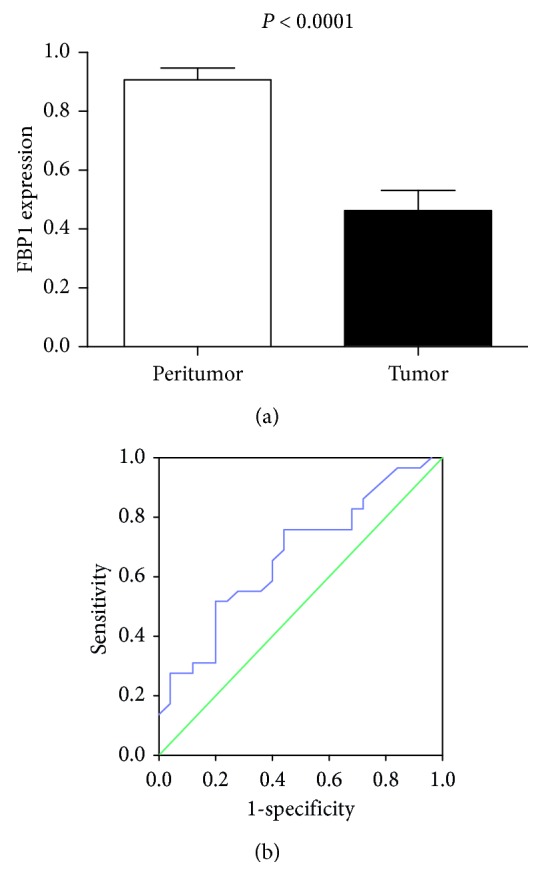
The relationship between ^18^F-FDG uptake and FBP1 expression in ccRCC. (a) FBP1 expression in ccRCC. The expression of FBP1 in ccRCC tissues (0.46 ± 0.07) was significantly lower than that in corresponding peritumor tissues (0.91 ± 0.04) (*P* < 0.0001). (b) Receiver-operating characteristic curve analysis of SUVmax in primary tumor to predict FBP1 expression in ccRCC. With an SUVmax of 3.35 as the optimal value, sensitivity and specificity for prediction FBP1 expression were 88.9% and 58.3%, respectively. The area under the receiver-operating characteristic curve was 0.67 (95% confidence interval: 0.53–0.81; *P*=0.032).

High FBP1 expression was detected in 90.7% (49/54) of the peritumor tissues and 46.3% (25/54) of the tumor tissues. As shown in Figure 1(a), the mean score of FBP1 in ccRCC tissues (0.46 ± 0.07) was significantly lower than corresponding peritumor tissues (0.91 ± 0.04) (*P* < 0.0001). The relationship between FBP1 expression and clinicopathological characteristics is shown in Table 2. No significant differences in FBP1 expression were found in terms of age, sex, or lymph node metastasis. However, FBP1 expression levels differed in tumor grade (*P*=0.023), SUVmax (*P*=0.020), and tumor size (*P*=0.003) (Table 2).

Next, we sought to determine the SUVmax threshold for optimal differentiation between high and low FBP1 expression groups. Receiver-operating characteristic curve analysis revealed that the highest accuracy (66.7%) was obtained with an SUVmax cutoff of 3.55 and that the area under the curve was 0.67 ± 0.07. Sensitivity and specificity for the prediction of FBP1 expression were 88.9% (22/29) and 58.3% (14/25), respectively (Figure 1(b)).

### 3.3. Relationship between SUVmax, FBP1 Expression and Tumor Differentiation

**Figure 2:**
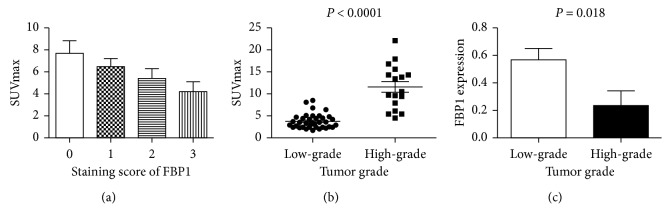
The relationship between FBP1 expression, SUVmax, and tumor grade in patients with ccRCC. (a) The relationship between SUVmax and the staining score of FBP1 in ccRCC. When the score of FBP1 staining was 0, 1, 2, or 3, SUVmax of ccRCC was 7.7 ± 5.8, 6.5 ± 1.3, 5.4 ± 2.8, and 4.2 ± 3.2, respectively. (b) SUVmax analysis in high-grade ccRCC and low-grade ccRCC. SUVmax was higher in patients with high-grade ccRCC (mean, 11.6 ± 5.0) than in those with low-grade ccRCC (mean, 3.8 ± 1.6, *P* < 0.001). (c) FBP1 expression levels in patients with high-grade ccRCC and low-grade ccRCC. FBP1 expression was significantly lower in patients with high-grade ccRCC (mean, 0.23 ± 0.1) than in those with low-grade ccRCC (mean, 0.57 ± 0.08; *P*=0.018).

We sought to further explore the association between FBP1 expression and ^18^F-FDG uptake in ccRCC. SUVmax of primary ccRCCs was 7.7 ± 5.8, 6.5 ± 1.3, 5.4 ± 2.8, and 4.2 ± 3.2 for 0, 1, 2, or 3 FBP1 staining scores, respectively. There was an inverse relationship between FBP1 expression and SUVmax in ccRCC patients (Spearman correlation coefficient, −0.295; *P*=0.031) (Figure 2(a)), indicating that the higher the FBP1 expression, the lower the SUVmax. Additionally, we investigated the association between SUVmax and tumor grade and found that patients with high-grade ccRCC demonstrated a higher SUVmax (11.6 ± 5.0) compared with patients with low-grade ccRCC (SUVmax, 3.8 ± 1.6; *P* < 0.001) (Figure 2(b)). High ^18^F-FDG uptake was observed in 59.5% (22/37) of patients with low-grade ccRCC and 100% (17/17) of patients with high-grade ccRCC. Furthermore, patients with high-grade ccRCC exhibited lower FBP1 expression (mean, 0.23 ± 0.1) compared with low-grade ccRCC patients (mean, 0.57 ± 0.08; *P*=0.018) (Figure 2(c)). Increased FBP1 expression was found in 56.8% (21/37) of patients with low-grade ccRCC and 23.5% (4/17) of patients with high-grade ccRCC.

### 3.4. Association of ^18^F-FDG Uptake and FBP1 Expression with GLUT1 and HK2 Expression

**Figure 3:**
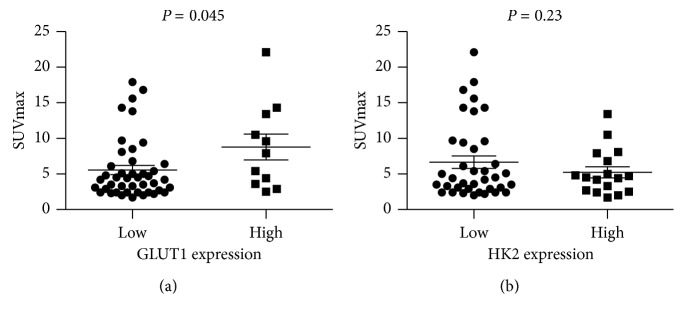
The relationship between SUVmax and the expression of GLUT1 and HK2 in primary ccRCC tumors. (a) The relationship between SUVmax and GLUT1 expression in primary ccRCC tumors. The SUVmax of ccRCC in the high GLUT1 expression group (mean, 8.78 ± 6.04) was significantly higher than that in the low GLUT1 expression group (mean, 5.55 ± 4.23; *P*=0.045). (b) The relationship between SUVmax and HK2 expression in primary ccRCC tumors. There was no significant difference in SUVmax according to HK2 expression groups (*P*=0.23).

We observed a positive association between SUVmax and GLUT1 expression in ccRCC (Figure 3(a)) but not between SUVmax and HK2 expression (Figure 3(b)). The SUVmax was significantly higher in the high GLUT1 expression group (mean, 8.78 ± 6.04) than in the low GLUT1 expression group (mean, 5.55 ± 4.23; P = 0.045). In addition, a significant inverse association was found between GLUT1 expression and FBP1 expression (*P*=0.036) but not between HK2 expression and FBP1 expression (*P*=0.609) (Table 3). These results suggest that FBP1 decreases ^18^F-FDG uptake, possibly by regulating GLUT1 expression.

## 4. Discussion

^18^F-FDG PET/CT has been widely used for diagnosis, staging, and monitoring of therapeutic response in many malignant tumors [8–10, 20–22]. However, ^18^F-FDG PET/CT has not been recommended for routine evaluation of renal tumors because of false-negative cases. However, the underlying mechanism for relatively low ^18^F-FDG uptake in some ccRCC remains unclear. Our study showed that SUVmax was significantly higher in ccRCCs with low FBP1 expression compared with high FBP1 expression, indicating that ^18^F-FDG uptake might reflect FBP1 expression levels in patients with ccRCC.

The kidney is one of the major organs of gluconeogenesis which is only next to the liver. Our previous study showed that the key enzyme of gluconeogenesis, FBP1, was downregulated and played an important role in regulating ^18^F-FDG uptake in hepatocellular carcinoma [18]. Similar to the expression of FBP1 in hepatocellular carcinoma [18], our current study showed that FBP1 was also decreased in ccRCC tissues compared with peritumor tissues, which is consistent with previous studies [17]. These results indicate that FBP1 is suppressed in some patients with ccRCC and that FBP1 might play a critical role during tumor development of ccRCC and possibly can serve as a novel molecular biomarker for ccRCC. Our study also showed that FBP1 expression was associated with tumor size, tumor grade, and SUVmax; thus, tumor size, tumor grade, and SUVmax may be good predictors for FBP1 in ccRCC.

We also assessed the association between the expression of FBP1, ^18^F-FDG uptake tumor, and tumor differentiation grade in patients with ccRCC. In ccRCC specimens, we found that SUVmax was significantly higher in high-grade ccRCC than in low-grade ccRCC; this finding is consistent with previous studies [15]. Furthermore, we showed that FBP1 expression was significantly lower in high-grade ccRCC tumors than in low-grade tumors. Consistent with these results, an inverse association was observed between FBP1 expression and SUVmax. These results suggest that the low uptake of ^18^F-FDG in some ccRCCs was due to the high FBP1 expression.

^18^F-FDG uptake in malignant tumors depends mainly on hexokinases and glucose transporters, both of which are overexpressed in many malignant tumors [10]. Several previous studies have found that ^18^F-FDG uptake in RCC was associated with the expression of GLUT1 [23–26]. Likewise, our results showed that there was a positive association between SUVmax and GLUT1 expression in ccRCC. Our results also showed that there was an inverse association between GLUT1 expression and FBP1 expression, whereas HK2 expression was not associated with FBP1 expression. In addition, our findings showed that the higher the FBP1 expression in ccRCC patients, the lower the SUVmax. Consistent with our clinical data, a previous study reported that *in vitro* overexpression of FBP1 in RCC cell lines led to a significant decrease in 18F-FDG uptake and GLUT1 expression [17]. Thus, we suggest the hypothesis that FBP1 might decrease ^18^F-FDG uptake via the downregulation of GLUT1 expression in ccRCC. Of course, the specific mechanism on how FBP1 affects ^18^F-FDG uptake in ccRCC should be further confirmed.

FBP1 expression was lower in ccRCC patients than in patients with normal kidneys. In addition, the expression levels of FBP1 varied widely in patients with different grades of ccRCC. FBP1 expression in patients with high-grade ccRCC was lower compared with low-grade ccRCC patients, whereas SUVmax was significantly higher in patients with high-grade ccRCC; thus, ^18^F-FDG PET/CT detected poorly differentiated RCCs with high sensitivity. In contrast, FBP1 expression was relatively high in patients with low-grade ccRCC, which corresponded to low ^18^F-FDG uptake. Consequently, ^18^F-FDG PET exhibited relatively low sensitivity for the detection of low-grade ccRCC, which has been noted in previous studies [15].

Our study had some limitations. First, the sample size of this study was small because our study was conducted at only one center. Second, there was unavoidable selection bias because our study was a retrospective study. Third, the kidneys are sites of physiological radioactive urine collection, and it is difficult to distinguish pathological uptake from the physiological radioactive urine collection in some cases. Furthermore, though the correlation between FBP1 and SUVmax is present, it is not very high. Large prospective studies that include more sample sizes and academic centers are needed to confirm our results in the further.

In conclusion, we found that in patients with ccRCC, ^18^F-FDG uptake and tumor grade (from low-grade to high-grade) were both inversely correlated with FBP1 expression. Our study also showed that FBP1 expression was correlated with tumor size, tumor grade, and SUVmax. FBP1 appears to inhibit ^18^F-FDG uptake via regulating GLUT1. These results suggest the underlying mechanisms of low ^18^F-FDG uptake in partial ccRCC. These results also lay the foundation for the development of a new imaging agent in the diagnosis of ccRCC with high sensitivity.

## Figures and Tables

**Table 1:** Patients and tumor characteristics.

Characteristics	No. of patients
*Sex*	
Male	37
Female	17
*Age (y)*	
Mean ± SD	58.9 ± 10.4
Range	31–82
*Tumor size (cm)*	
Mean ± SD	6.47 ± 4.01
Range	1.2–20
*Lymph node metastasis*	
Negative	43
Positive	11
*Tumor grade*	
Low	37
High	17
*SUVmax*	
Mean ± SD	6.21 ± 4.78
Range	1.7–22.1
*FBP1 expression*	
Low	29
High	25

**Table 2:** Relationship between FBP1 expression and clinicopathological characteristics of ccRCC patients.

Variable	Total	FBP1 expression		*P*
		Low	High	
*Sex*				
Male	37	20	17	0.939
Female	17	9	8	
*Age (y)*				
<60	25	14	11	0.753
≥60	29	15	14	
*Tumor size (cm)*				
≤7	34	13	21	0.003
>7	20	16	4	
*Lymph node metastasis*				
Negative	43	22	21	0.459
Positive	11	7	4	
*Tumor grade*				
Low	37	16	21	0.023
High	17	13	4	
*Mean SUVmax*		7.56 ± 5.57	4.66 ± 3.08	0.02

**Table 3:** Relationship between FBP1 expression and expression of GLUT1 and HK2.

		FBP1 expression		*P*
		Low	High	
GLUT1 expression	Low	20	23	0.036
	High	9	2	
HK2 expression	Low	19	18	0.609
	High	10	7	

## Data Availability

The data used to support the findings of this study are available from the corresponding author upon request.
